# Prevalence of HIV-Associated Metabolic Abnormalities among Patients Taking First-Line Antiretroviral Therapy in Uganda

**DOI:** 10.5402/2012/960178

**Published:** 2012-08-23

**Authors:** Bernard Omech, Joseph Sempa, Barbara Castelnuovo, Kenneth Opio, Marcel Otim, Harriet Mayanja-Kizza, Robert Colebunders, Yukari C. Manabe

**Affiliations:** ^1^School of Medicine, Makerere University College of Health Sciences, P.O. Box 7072, Kampala, Uganda; ^2^Research Department, Infectious Diseases Institute, Makerere University College of Health Sciences, P.O. Box 22418, Kampala, Uganda; ^3^Institute of Tropical Medicine, Nationalestraat 155, University of Antwerp, Antwerp, Belgium; ^4^Division of Infectious Diseases, Department of Medicine, Johns Hopkins University School of Medicine, Baltimore, MD, USA

## Abstract

*Introduction*. While the introduction of highly active antiretroviral therapy decreased HIV-related morbidity and mortality rates in the sub-Saharan Africa, a subsequent increase in metabolic abnormalities has been observed. We sought to determine the prevalence of HIV-associated metabolic abnormalities among patients on first-line antiretroviral therapy (ART) in an ART clinic in Kampala, Uganda. *Methods*. Four hundred forty-two consecutive patients on first-line ART for at least 12 months were screened for eligibility in a cross-sectional study, and 423 were enrolled. Pre-ART patient characteristics were abstracted from medical charts, examinations included anthropometric measurement and physical assessment for lipodystrophy. *Results*. The prevalence of hyperglycemia and dyslipidemia was 16.3% (69/423) and 81.5% (345/423), respectively. Prevalence of dyslipidemia between stavudine- and zidovudine-based regimens (91% versus 72%; *P* < 0.001). Being on stavudine (aOR 4.79, 95%, 2.45–9.38) and peak body weight (aOR 1.44, 95% CI 1.05–1.97) were independent risk factors for dylipidemia. Stavudine (aOR 0.50, 95% CI  0.27–0.93) use was associated with lower risk for hyperglycemia while, and older age (aOR 1.31, 95% CI 1.11–1.56) and having a family history of DM (aOR 2.18, 95% CI 1.10–4.34) were independent risk factors for hyperglycemia. *Conclusions*. HIV-associated metabolic complications were prevalent among patients on thymidine analogue-containing ART regimens. Screening for lipid and glucose abnormalities should be considered in ART patients because of cardiovascular risks.

## 1. Introduction

As antiretroviral therapy (ART) coverage in sub-Saharan African (SSA) populations continue to increase, [1] deaths from HIV/AIDS has dramatically declined in the past decade [2]. In SSA and other resource limited settings (RLS), where stavudine-containing ART regimens have been extensively used, a wide range of disfiguring body fat changes and metabolic abnormalities have been reported [3–5]. Stavudine and zidovudine are both thymidine nucleoside analogues used in combination with lamivudine and either nevirapine or efavirenz as first-line therapy of HIV in most RLS. After a recent WHO recommendation against use of stavudine-containing regimens, [6] stavudine has been largely discontinued, but a large proportion of patients remain on zidovudine-containing regimens in Uganda and other SSA due to cost considerations. The mechanisms of these metabolic complications remain largely unclear, but several studies suggest that HIV itself, cytokine disregulation [7, 8], and the thymidine nucleosides-induced mitochondrial toxicity contribute to these abnormalities [9]. Epidemiological data on these metabolic abnormalities and risk of cardiovascular morbidity are still limited in SSA. A few cross-sectional studies in the region have concentrated mainly on stavudine-containing regimens and little is reported on zidovudine-containing regimens [3, 5, 10]. In this study we describe the metabolic abnormalities observed in patients on first-line ART in an urban outpatient infectious diseases clinic in Kampala, Uganda on ART for at least 12 months.

## 2. Materials and Methods

### 2.1. Recruitment and Procedures

Between May and August 2008, patients were enrolled for the study at the Adult Infectious Disease Clinic at the Infectious Diseases Institute (IDI) in Kampala, Uganda. We enrolled patients aged 18 years and above, on first line ART for at least 12 months with an adherence level by self-report of ≥95% and who gave informed consent. After fasting for at least 8 hours, blood was drawn for glucose, lipid profile and CD4 count. Persons were excluded if their ART had ever been switched or substituted, they were diabetic prior to ART initiation, had index dyslipidemias, or used lipid-lowering drugs, corticosteroids, or oral contraceptives pills. A trained research assistant obtained a medical history from the patient and abstracted clinical information from the patient's chart including the date of ART start, pre-ART CD4 count, WHO clinical stage, body weight at ART start, current CD4 count, and peak body weight while on ART. A clinician, masked to the patient's ART regimen, performed a physical examination and anthropometric measurements, including the determination of blood pressure (BP) and waist/hip circumferences. A patient was considered hypertensive, when the systolic blood pressure was ≥140 mmHg or diastolic BP was ≥90 mmHg on more than two occasions. All patients were assessed for evidence of subcutaneous fat loss or fat accumulation. Features suggestive of fat loss in the face included sunken eyes, prominent zygoma, sunken cheeks, and in the upper and lower limbs included prominent veins, subcutaneous fat wasting especially in the arms. Features of fat accumulation were the presence of excessive abdominal fat compared to peripheral fat (after excluding organomegaly and intra-abdominal masses as assessed by palpation), buffalo humps and lipomata on any part of the body. 

CD4 cell count was done using BD FACSCalibur (Becton Dickinson, Mountain View, CA, USA). Serum lipid assays were batched and run by an automated enzyme-based chemistry analyzer (Konelab 30 system, Thermo Electron Corporation, Finland), with quality controls done at the beginning of each batched run. Ten duplicate samples from each batch were analyzed in a separate laboratory within the National Referral Hospital complex as an external quality check. Finger prick blood for glucose was done on an automated one touch analyzer (Lifescan. Inc, United Kingdom). Participants with fasting blood sugar ≥7 mM were asked to return for a repeat test to confirm diabetes mellitus (DM) at the next convenient date. Those confirmed with DM were referred to a specialist at the diabetic clinic for further management. 

### 2.2. Operational Definitions

The international classification system recommended by the World Health Organization (WHO) and the National Cholesterol Education Program (NCEP) for glucose and lipid profiles [11–13] were used (Table 1).

### 2.3. Statistical Analysis

Data was cleaned and entered in Epidata version 3.1 and exported to STATA version 10.0 for analysis. At univariate level all variables were compared according to ART regimen (stavudine- or zidovudine-containing regimen). Categorical variables were compared using a Chi square test and a *P* value was obtained. A *P* value <0.05 was considered statistically significant. Continuous variables were compared using a Wilcoxon rank-sum test, a nonparametric test. 

At bivariate level, all predictor variables including ART regimen were compared with lipid and glucose levels: both were dichotomized into abnormal and normal levels. Study participants were categorized as having abnormal glucose levels if they had ≥6.11 mmol/L, while those who were classified as having abnormal lipid levels had either total cholesterol ≥5.17 mmol/L or ldl-cholesterol ≥3.36 mmol/L or hdl-cholesterol ≥1.53 mmol/L or triglycerides ≥2.26 mmol/L. Odds ratio was used as the measure of association, a 95% confidence interval (CI) was obtained, and a *P* value was used to show statistical significance. Variables with a *P* value < 0.20, and those which had clinical significance were used in the multivariate model to identify factors associated with abnormal lipid and glucose levels. The final model was tested for suitability in predicting the outcome using the Hosmer-Lemeshow test which a variant of Chi square test and the model was deemed good if *P* < 0.05. The study was approved by Makerere University Department of Medicine, Faculty of Medicine Research and Ethics Board.

## 3. Results

### 3.1. Patient Characteristics

Four hundred forty-two patients on ART were enrolled into the study between May–August, 2008. Nineteen patients had incomplete chart records and were excluded from the analysis. Of the 423 patients analyzed, 60% (255/423) were women. The median (IQR) age of the study participants was 39 years (34–44), and the median (IQR) initial CD4 counts was 90 cells/*μ*L (33–168). Two hundred and eight (49%) were started on stavudine plus lamivudine plus nevirapine and 215 (51%) on zidovudine plus lamivudine plus efavirenz (*n* = 203) or nevirapine (*n* = 12).

Results presented in Table 2 show the characteristics of the patients initiated on stavudine- and zidovudine-based regimens. Compared with the patients on zidovudine patients on stavudine were more frequently male, had more often a family history of hypertension, had been on ART for a shorter time, and had a higher median CD4 count at enrollment. Physician-assessed lipodystrophy was significantly higher in patients on stavudine with a greater median waist-to-hip ratio in the stavudine group. Patients on stavudine also had a higher median systolic blood pressure and overall hypertension. 

### 3.2. Dyslipidemia

The overall prevalence of patients with dyslipidemia was 81.6% (345/423). The majority (60%, 253/423), had undesirable high-density lipoprotein C (HDL-C), defined as <1.53 mmol/L; 39% (165/423) had high total cholesterol (TC), 24% (100/423), high triglycerides (TG), and 20% (83/423) elevated low-density lipoprotein C (LDL-C) (Table 3). The proportion of patients with borderline and overt dyslipidemia on stavudine compared to AZT was higher (91%, 22% versus 72%, 16% resp.). 

### 3.3. Hyperglycemia

The overall prevalence of hyperglycemia (defined as >6.11 mmol/L) was (69/423, 16.3%) of which 63.8% had borderline hyperglycemia, and 36.2% had overt diabetes mellitus (defined as >6.94 mmol/L). The proportion of patients with borderline hyperglycemia and overt diabetes mellitus on stavudine compared to AZT were similar (11%, 3% versus 18%, 5% resp.). 

### 3.4. Multivariate Analysis

At bivariate analysis, use of stavudine (odds ratio (OR) 4.18, 95% CI 2.37–7.37, *P* < 0.001), and lipodystrophy (OR 2.77, 95% CI 1.28–5.99, *P* = 0.01) were more likely to be associated with dyslipidemia compared to use the zidovudine; a peak body weight >65 kgs was also more likely to be associated with dyslipidemia (OR 1.35, 95% CI 1.03–1.75 *P* = 0.027); family history of hypertension and diabetes mellitus increased the odds of being diagnosed with dyslipidemia (OR 1.75, 95% CI 1.02–2.98; *P* = 0.040 and OR 2.29, 95% CI 1.05–4.97, *P* = 0.037), respectively (Table 4). Factors that were associated with hyperglycemia at bivariate level were a 5-year increase in age (OR 1.32, 95% CI 1.14–1.54, *P* < 0.001), and an abnormal waist-to-hip ratio (OR 1.97, CI 1.01–3.84, *P* = 0.046) (Table 5).

At multivariable analysis, the use of stavudine and peak body weight were independently associated with dyslipidemia (adjusted odds ratio (aOR) 4.79, 95% CI 2.45–9.38; *P* < 0.001 and aOR 1.44, CI 1.05–1.97; *P* = 0.023) (Table 4). We repeated the analysis excluding patients with borderline lipid abnormalities and we found that the only factor independently associated with high lipid levels was obesity at enrollment (aOR 3.2, 95% CI 1.22–8.27; *P* = 0.018) (results not shown).

The use of stavudine had a protective effect on developing hyperglycemia (aOR 0.50, 95% CI 0.27–0.93; *P* = 0.028); a 5-year increase in age increased the odds of developing hyperglycemia (aOR 1.31, 95% CI 1.11–1.56; *P* = 0.002) and; family history of diabetes mellitus increased the odds of having hyperglycemia (aOR 2.18, 95% CI 1.10–4.34; *P* = 0.026) (see Table 5).

## 4. Discussion

Routine baseline fasting glucose and lipid profile are not usually measured in HIV-infected patients initiating ART in resource-limited settings like Uganda, due to cost. In this report, the overall prevalence of fasting dyslipidemia of 81.5% is the highest among published reports so far in SSA population. We observed a higher prevalence of dyslipidemia among patients on stavudine-containing regimens compared with zidovudine-containing regimen. The majority of these lipid abnormalities were due to borderline decreased levels of cardioprotective HDL-C in both categories of ART regimens. We observed a higher prevalence of an atherogenic lipid profile in patients on stavudine compared with zidovudine, TC (44.7% versus 33.7%), LDL-C (24.5% versus 14.9%), and TG (28.4% versus 19.1%), but AZT-based regimens also had notable rates of dyslipidemia. A similar study in an urban setting in Kenya among patients on stavudine-based regimens reported an overall prevalence of dyslipidemia of 63.1% [10]. In a retrospective study in a rural setting in eastern Uganda an even lower prevalence of dyslipidemia (10–41%) was noted but ART regimens in the latter study were not constant during followup [14].

 Fasting hyperglycemia was observed in 14.4% of our patients (4% meeting the criteria of diabetes mellitus), with no significant differences between the ART regimens. This is a slightly lower prevalence than that reported from Rwanda (16%–18%) [5] and Kenya (20.7%) [14] in patients on a stavudine regimen. 

 There were several differences observed between the patients initiated on stavudine- and zidovudine-containing regimens at enrollment. Patients on stavudine-containing regimens on average had a shorter duration of therapy compared to zidovudine, probably because of toxicities related to stavudine (peripheral neuropathy) [15] and nevirapine (liver toxicity and rash) prompting early switch of therapy. We found a higher prevalence of hypertension among patients on a stavudine-containing compared to a zidovudine-containing regimen (16% versus 7%). Moreover lipodystrophy was more prevalent in stavudine-based patients compared to zidovudine (35.8% versus 6.7%). In spite of lack of a uniform definition of lipodystrophy across the literature, our findings are similar to those observed in patients on stavudine-containing regimens in the region [5, 16]. Previous studies have documented an association between ART-associated lipodystrophy and hypertension but the explanation for this remains unclear [17]. 

 Thymidine nucleosides, especially stavudine, are associated with mitochondrial toxicity and reduced clearance of free fatty acids and triglycerides, which is currently the most favored hypothesis to partly explain the atherogenic lipid profile and body fat redistribution in HIV-infected patients [7–9]. In addition, interactions of nonnucleoside drug combinations on lipid metabolism may be significant in a setting where stavudine was most often given with nevirapine. Nevirapine was reported to have a more favorable lipoprotein profile (causing elevation of the HDL-C) compared with efavirenz [18]. 

 We identified two independent factors associated with dyslipidemia, being on a stavudine-containing regimen and a peak body weight of >65 kgs during ART. The majority of our patients were from an urban setting, and compared with individuals from rural communities, they are more likely to live a sedentary life, and to eat unhealthy diets which may be confounders in this study. Age and ART duration which have been reported in another study, [19] were not significantly associated with dyslipidemia possibly because we only enrolled patients who were already on therapy for at least one year. Lipodystrophy was significantly associated with dyslipidemia at bivariate analysis, although it was not significant when adjusted for other factors. Age over 40 years was independently associated with hyperglycemia. However, at subanalysis when patients with borderline glucose levels were excluded, being overweight at assessment and obese at enrollment (by BMI) were independently associated with overt diabetes mellitus whereas higher median CD4 (>100 cells/*μ*L) at initiation of ART was protective (data not shown). Again, aging and urban lifestyle are likely to play a significant role in the development of insulin resistance and diabetes mellitus in HIV-infected patients on ART.

 The consequences of these HIV-associated metabolic disturbances in an African population are still unclear. However from DAD study in Europe and North American populations, a significant risk of cardiovascular morbidity was reported [20]. The major limitations of this study are its cross-sectional design, nonrandomized treatment allocations and a subjective clinical assessment of lipodystrophy. Allocation to fixed regimen occurred in RLS in sub-Saharan Africa where the relative risk of thymidine analogues compared to NNRTI could not be separated. We also did not perform a glucose tolerance test. Therefore we may have underestimated the number of patients with impaired glucose metabolism. 

Although increased access to relatively cheaper generic fixed-drug combinations of ART have dramatically improved the outcome of patient with HIV infection in Africa, the problem of the increasing prevalence of dyslipidemia, hyperglycemia and body fat changes associated with the commonly available ART regimens should be addressed. Coupled with the adoption of western diets and sedentary lifestyle in our urban population, we are likely to see a steady rise in atherosclerotic diseases in patients on long-term ART. Mutimura and colleagues in Rwanda have shown in a clinical trial that simple but regular exercise can be beneficial in alleviating lipodystrophy and improving metabolic abnormalities, which can be adopted in an ART package of HIV-infected patients in RLS [21]. Our study justifies the Ministry of Health action in Uganda to phase out stavudine-use from first-line ART drug list but our study shows that zidovudine contributes to metabolic abnormalities as well. All HIV-infected patients on thymidine analogues should be screened for glucose and lipid abnormalities. Furthermore, prospective studies to evaluate long term effect of these metabolic disturbances on cardiovascular health are needed. 

## Figures and Tables

**Table 1:** Lipid and glucose international classification recommended by the World Health Organization.

Classification	TC (mmol/L)	LDL (mmol/L)	HDL (mmol/L)	TG (mmol/L)	GLU (mmol/L)
Desirable	<5.17	<3.36	≥1.53	<2.26	<6.11
Borderline	5.17–6.18	3.36–4.11	0.91–1.53	2.26–4.50	6.11–6.94
High	>6.21	>4.14		≥4.50	>6.94
Low			<0.91		

TC: total cholesterol; LDL: low-density lipoprotein; HDL: high-density lipoprotein; GLU: Glucose.

**Table 2:** Characteristics of patients initiated on stavudine- and zidovudine-based regimens.

Characteristics	Stavudine (*N* = 208)	Zidovudine (*N* = 215)	*P* value
Gender, male. *N* (%)	72 (34.6)	96 (44.6)	0.035
Age, (5 years), median (IQR)	38 (33–43)	39 (34–46)	0.131
ART duration (years), median (IQR)	2.5 (1.8–2.9)	2.7 (1.8–3.3)	0.016
Initial CD4+ counts, median (IQR)	94 (30.5–171)	89 (38–166)	0.888
Initial body weight (Kg), median (IQR)	53.8 (48.3–61.5)	54 (48–60)	0.784
Peak body weight, median (IQR)	63 (57–71)	63 (57–68)	0.387
BMI >25 at study enrolment	57 (27.4)	56 (26.1)	0.752
Systolic blood pressure (mmHg), median (IQR)	120 (110–130)	110 (110–120)	<0.001
Hypertension, *N* (%)*	33 (15.9)	16 (7.4)	0.007
CD4 at study enrolment >200 cells/*μ*L	175 (85.0)	153 (72.5)	0.002
Abnormal waist to hip ratio^+^	0.94 (0.88–0.99)	0.91 (0.88–0.95)	<0.001
Family history of hypertension, *N* (%)	90 (43.7)	66 (30.7)	0.006
Family history diabetes mellitus, *N* (%)	42 (20.4)	34 (15.8)	0.223
Lipodystrophy, *N* (%)	74 (35.8)	14 (6.7)	<0.001
WHO stage III and IV, *N* (%)	151 (72.6)	166 (77.2)	0.274

*N*: number; IQR: interquartile range; WHO: World Health Organization; ART: antiretroviral treatment; BMI: body mass index.

*Hypertension defined as systolic blood pressure ≥140 mmHg or diastolic blood pressure ≥90 mmHg.

^+^Waist to hip ratio was categorized into abnormal if the value was >0.94 for males and >0.8 for females.

**Table 3:** Prevalence of dyslipidemia and hyperglycemia in HIV-infected patients according to stavudine- and zidovudine-containing regimens.

	Stavudine	Zidovudine	Total	
Lipid abnormalities	*N* = 208	*N* = 215	*N* = 423	*P* value
	*N* (%)	*N* (%)	*N* (%)	
Total cholesterol				
Borderline	58 (27.9)	48 (22.3)	106 (25.1)	0.018
High	35 (16.8)	24 (11.2)	59 (14.0)	
LDL-cholesterol				
Borderline	29 (13.9)	17 (7.9)	46 (10.9)	0.013
High	22 (10.6)	15 (7.0)	37 (44.6)	
Triglycerides				
Borderline	40 (19.2)	31 (14.4)	71 (16.8)	0.025
High	19 (9.1)	10 (4.7)	29 (6.9)	
HDL-cholesterol				
Borderline	147 (70.7)	87 (40.5)	234 (55.3)	<0.001
Low	12 (5.8)	7 (3.3)	19 (4.5)	
Dyslipidemia				
Borderline	190 (91.4)	155 (72.1)	345 (81.6)	<0.001
Overt	46 (22.1)	34 (15.8)	80 (18.9)	
Hyperglycemia				
Borderline	23 (11.1)	38 (17.7)	61 (14.4)	
Overt	6 (2.9)	11 (5.1)	17 (4.0)	

TC: total cholesterol; LDL: low-density lipoprotein; HDL: high-density lipoprotein.

**Table 4:** Risk factors for borderline dyslipidemia.

Characteristics	Odds ratio	95% CI	*P* value	Adjusted odds ratio	95% CI	*P* value
Male gender	0.92	0.56–1.50	0.734			
Stavudine-based ART regimen	4.18	2.37–7.37	<0.001	4.79	2.45–9.38	<0.001
Age	1.03	0.89–1.19	0.696			
ART duration	1.24	0.95–1.63	0.113			
Initial CD4 counts per 50 cells/*μ*L increase	0.99	0.86–1.15	0.932			
ART start body weight	1.02	0.90–1.05	0.115			
Peak body weight >65 kgs	1.35	1.03–1.75	0.027	1.44	1.05–1.97	0.023
Enrolment BMI <24	1.26	0.77–2.27	0.423			
Systolic blood pressure >150 mm/Hg	1.00	0.99–1.02	0.443	0.99	0.97–1.06	0.153
Enrolment CD4 count >200 cells/*μ*L	0.90	0.49–1.64	0.734			
Abnormal waist to hip ratio^+^	1.23	0.73–2.07	0.446	1.14	0.63–2.05	0.673
Family history of hypertension	1.75	1.02–2.98	0.04	1.44	0.79–2.64	0.235
Family history of diabetes mellitus	2.29	1.05–4.97	0.037			
Lipodystrophy	2.77	1.28–5.99	0.01	1.60	0.61–4.21	0.338
WHO stage III and IV	1.21	0.71–2.08	0.487			

CI: confidence interval; ART: antiretroviral treatment; BMI: body mass index; WHO: World Health Organization.

^+^WHR defined abnormal if >0.94 for males and >0.8 for females.

**Table 5:** Risk factors for borderline hyperglycemia.

Characteristics	Odds ratio	95% CI	*P* value	Adjusted odds ratio	95% CI	*P* value
Male gender	0.76	0.44–1.33	0.336	0.93	0.41–2.12	0.855
Stavudine-based ART regimen	0.58	0.33–1.01	0.055	0.50	0.27–0.93	0.028
Age	1.32*	1.14–1.54	<0.001	1.31	1.11–1.56	0.002
ART duration	1.09	0.82–1.46	0.550			
Initial CD4 counts per 50 cells/*μ*L increase	0.94	0.80–1.11	0.463			
ART start body weight	1.00	0.97–1.03	0.95			
Peak body weight >65 kgs	1.00	0.92–1.09	0.957			
Enrolment BMI <24	1.00	1.00–1.02	0.213			
Systolic blood pressure >150 mm/Hg	1.02	1.00– 1.03	0.05	1.01	0.90–1.03	0.146
Enrolment CD4 count >200 cells/*μ*L	1.06	0.55–2.05	0.86			
Abnormal waist to hip ratio^+^	1.97	1.01–3.84	0.046	1.73	0.67–4.46	0.255
Family history of hypertension	0.99	0.57–1.73	0.972			
Family history of diabetes mellitus	1.71	0.91–3.22	0.095	2.18	1.10–4.34	0.026
Lipodystrophy	0.98	0.56–1.89	0.946			
WHO stage III and IV	1.00	0.54–1.85	0.991			

CI: confidence interval; ART: antiretroviral treatment; BMI: body mass index; WHO: World Health Organization.

^+^WHR defined abnormal if >0.94 for males and >0.8 for females.

*Denote that age as a variable was supposed to be interpreted as a 5-year increase as interpreted in the text.
